# Direct and indirect aggression and victimization in adolescents - associations with the development of psychological difficulties

**DOI:** 10.1186/s40359-014-0043-2

**Published:** 2014-10-12

**Authors:** Lars-Gunnar Lundh, Daiva Daukantaité, Margit Wångby-Lundh

**Affiliations:** Department of Psychology, Lund University, Box 213, 221 00 Lund, Sweden

**Keywords:** Direct aggression, Indirect aggression, Direct victimization, Indirect victimization, Emotional problems, Conduct problems, Longitudinal design, Bidirectional associations

## Abstract

**Background:**

Previous research has established that direct and indirect forms of aggression differ in their association with gender and type of psychological difficulties. One purpose of the present study was to test if the same applies to direct and indirect victimization. A second purpose was to study these associations not only cross-sectionally (as in most previous research) but also longitudinally. A third purpose was to test the hypotheses that there are prospective bidirectional associations not only between victimization and psychological difficulties (which has been shown in previous research), but also between aggression and psychological difficulties, and that direct and indirect forms of aggression and victimization show different associations with different types of psychological difficulties.

**Methods:**

The participants were a community sample of all students in two grades of regular school in a Swedish municipality who answered questionnaires as part of a two-wave longitudinal study with a one-year interval. The participants were 13–15 years old, and there were longitudinal data on 893 students, which represented 85% of all students. The cross-sectional associations were primarily tested by semi-partial correlations, and the longitudinal associations by hierarchical multiple regression.

**Results:**

The results corroborated the meaningfulness of differentiating not only between direct and indirect aggression but also between direct and indirect victimization. Boys reported being more victim to direct aggression, whereas girls reported being more victim to indirect aggression. Direct aggression predicted increased conduct problems in boys, whereas indirect aggression predicted increased conduct problems in girls, and conduct problems reciprocally predicted increased direct and indirect aggression. Indirect victimization showed prospective bidirectional associations with emotional symptoms and conduct problems, suggesting the potential development of vicious cycles of escalating problems in these areas.

**Conclusions:**

The present results indicate that direct and indirect aggression, as well as direct and indirect victimization, may have different roles in the development of psychological difficulties in young adolescents. Further, the demonstration of prospective bidirectional associations points to a possible mechanism for the development of psychological difficulties, that may be described in terms of dynamical systems theory. This has potential relevance both for the prevention and the treatment of psychopathology.

## Background

Aggressive behavior may be problematic both for those who behave aggressively, and for those who become victims of aggressive behavior. A more detailed understanding of these processes among children and adolescents are of particular interest, as both aggression and victimization are potentially formative factors in psychological development. Although previous research clearly indicates that both aggression (e.g., Card et al. 2008) and victimization (e.g., Hawker and Boulton 2000) are associated with various forms of distress and maladjustment, there is need for more detailed knowledge about the nature of these associations, both cross-sectionally and longitudinally. In particular, longitudinal knowledge of such relationships may be important for the prevention and treatment of various kinds of psychological difficulties.

A more detailed knowledge of these associations requires a differentiated view both of aggression and victimization, on the one hand, and various forms of psychological difficulties, on the other hand. If aggression is defined as any form of behavior that is intended to harm someone physically or psychologically (e.g., Berkowitz 1993), a number of distinctions can be made, as for example in terms of motivation (instrumental aggression vs. reactive aggression), means (physical versus relational aggression, or direct versus indirect aggression), and target (other-directed versus self-directed aggression). The present research focuses on the distinction between direct and indirect aggression, and asks if these two forms of aggression (both for aggressor and victim) are differently associated with various forms of psychological difficulties.

Psychological difficulties may also take many different forms, and the present research focuses on two broad categories of such difficulties: internalizing problems (emotional symptoms) and externalizing problems (conduct problems). The main question is how direct and indirect forms of aggression and victimization are associated with these different kinds of problems in the development of young adolescents, both as antecedents and as consequences.

### Direct and indirect aggression

Research on aggressive behavior in children and adolescents originally addressed direct, physical forms of aggression, documenting that these were more common among boys than among girls (e.g., Hyde 1984). In the 1980s and 1990s, a number of researchers started to study less direct forms of aggression, labelled either as “indirect aggression” (Björkqvist 1994) or “relational aggression” (Crick and Grotpeter 1995), and exemplified by behaviors like gossiping, spreading rumours, and social exclusion that may damage the victim’s self-esteem or social status. These types of aggressive behavior were not more common among boys; on the contrary, some studies even indicated that these less direct forms of aggression were more frequent among girls (e.g., Crick 1997), whereas other studies showed negligible gender differences. On the basis of a large meta-analysis, Card et al. (2008) concluded that although girls seem to engage significantly more in indirect aggression than boys, this difference is trivial in magnitude.

The validity of the distinction between direct and indirect aggression is supported by a number of factor-analytic studies (e.g., Crick and Grotpeter 1995; Grotpeter and Crick 1996; Vaillancourt et al. 2003), which show evidence of two aggression factors: one factor which includes both physical aggression and overt verbal attacks, and another factor that includes hurtful manipulation of relationships and damaging the target’s social position, often through indirect or covert means. The present paper follows Card et al. (2008) in labelling these two factors *direct* and *indirect* aggression. Although there is clear evidence for these two factors, Card et al. found the average correlation between direct and indirect aggression to be very high – and higher among boys than girls.

Previous research has shown that direct and indirect forms of aggression are differently associated with psychological problems. In Card et al.’s (2008) meta-analysis, direct aggression was more strongly associated with externalizing problems (conduct problems and ADHD problems), whereas indirect aggression was more strongly associated with internalizing problems (depression and anxiety). Interestingly, gender did not moderate these differential associations. Most of this research is cross-sectional, however, and little research has focused on the developmental associations of direct and indirect aggression with types of psychological difficulties by prospective, longitudinal designs.

### Direct and indirect victimization

Is the distinction between direct and indirect aggression also applicable to the experience of being the victim of aggressive behavior (i.e., victimization)? Although not much research has been addressed to this question, it is clearly possible to distinguish conceptually between the same behaviors from both the aggressor’s and the victim’s perspective. The relevant question is rather if direct and indirect victimization are empirically distinguishable in terms of their associations with other variables. Although victimization in general has been found to be clearly associated with psychological distress, both in cross-sectional studies (Hawker and Boulton 2000) and in prospective studies (Reijntjes et al. 2010; Reijntjes et al. 2011), it has been questioned (e.g., Card and Hodges 2008) whether the associations with psychological distress differ by victimization type. There is, however, some evidence in favour of a differentiation. First, several studies indicate that direct or physical forms of victimization are more common among boys, whereas indirect, relational forms of victimization are more common among girls (e.g., Nansel et al. 2001). Second, in a cross-sectional study, Prinstein et al. (2001) found that internalizing problems were more strongly related to relational victimization than to overt victimization, at least among girls. More research, however, is needed to clarify if there is a similar differentiation between direct and indirect victimization as between direct and indirect aggression, so that direct victimization is primarily associated with externalizing problems, whereas indirect victimization is primarily associated with internalizing problems.

### Longitudinal associations

How are direct and indirect aggression, direct and indirect victimization, and other psychological difficulties related developmentally? According to the specificity hypothesis of aggression (Ostrov 2008), children tend to retaliate in kind, which implies that physical victimization should be prospectively associated with physical aggression, and relational victimization with relational aggression. Testing these predictions in early childhood, Ostrov (2010) found that physical victimization was uniquely associated with increases in physical aggression over time, and that relational victimization was uniquely associated with increases in relational aggression over time. It is not known, however, whether this kind of specificity also applies to older children or adolescents.

Moreover, the question on developmental specificity can be widened to ask also if there are specific associations between various types of psychological difficulties and direct versus indirect forms of aggression and victimization. With regard to the longitudinal associations between aggression and other psychological difficulties, previous research has indicated that both direct (physical) and indirect (or relational) aggression is associated with an increase in social-psychological adjustment problems (Cleverley et al. 2012; Crick et al. 2006). There is little research reported, however, on whether direct and indirect aggression *differ* in their longitudinal associations with internalizing and externalizing types of psychological difficulties among adolescents.

Similarly, with regard to victimization, the results from several large prospective studies indicate that being victim of aggression from others is a risk factor for the development of emotional problems such as anxiety and depression (e.g., Bond et al. 2001; Sweeting et al. 2006; Zwierzynska et al. 2013), as well as conduct problems such as aggression and delinquency (e.g., Hanish and Guerra 2002; Kim et al. 2006). Again, however, there is little research reported on whether direct and indirect victimization differ in their longitudinal associations with internalizing and externalizing types of problems.

### Prospective reciprocal associations, and “vicious cycles”

Widening the longitudinal perspectives even further, a theoretically important topic concerns the possibility of prospective, *reciprocal* associations between aggression or victimization, on the one hand, and different types of psychological difficulties on the other. If such bidirectional associations can be documented, this may take us an important step towards delineating *mechanisms* for the development and aggravation of psychological difficulties. Basically, if such a bidirectional relationship can be established between two kinds of variables this means that they may enter into a self-generating “vicious cycle” where increases in the one variable lead to increases in the other, and vice versa – a process that can be described in terms of a dynamic system, where internal feedback processes lead to the emergence and stabilization of pathological patterns. This would be an example of what Bergman and Magnusson (1997) refer to as “problem gravitation” by means of interactions and two-way causality, which “push the child’s dynamic system into a stable state of multiple maladjustment” (p. 302).

Here there seems to be more research on victimization than on aggression. As pointed out in the previous section, the results from several large prospective studies indicate that being victim of aggression from others is a risk factor for the development of both emotional problems and conduct problems. At the same time, longitudinal studies also show that the *reverse* seems to be true: children with emotional problems (e.g., Hodges and Perry 1999; Sweeting et al. 2006) as well as children with conduct problems (e.g., Kim et al. 2006) have an increased risk of being bullied in childhood. This suggests that there may be a reciprocal bidirectional relation between the development of psychological problems in general and victimization by others.

Consistent with this assumption, Reintjes et al. (2010, 2011) in two meta-analyses – one on the prospective association between peer victimization and internalizing problems, and the other on the prospective association between peer victimization and externalizing problems – found support for such reciprocal associations. That is, peer victimization predicted subsequent increases in internalizing as well as externalizing problems, and internalizing and externalizing problems conversely predicted subsequent increases in peer victimization. As Reintjes et al. (2010, 2011) point out, this suggests the existence of escalating “vicious cycles” of victimization and internalizing as well as externalizing problems, where increased victimization leads to increases in various kinds of psychological problems, which in turn may lead to further increases in victimization.

Reijntjes et al. (2010, 2011) did not, however, separate indirect and direct victimization in their analyses. To test the hypothesis of bidirectional specificity, there is a need to study also if indirect victimization is specifically (and reciprocally) associated with internalizing problems, whereas direct victimization is specifically (and reciprocally) associated with externalizing problems. Further, there seems to be a lack of research on the question whether similar prospective reciprocal associations also exist between direct and indirect aggression, on the one hand, and different types of psychological difficulties on the other.

### The aims of the present study

To summarize, the present research addresses a number of questions about the specificity of direct and indirect aggression and victimization in their associations with various forms of psychological difficulties. Most of these questions have not been studied, or have been insufficiently studied, in previous research on young adolescents. These questions can be divided into three categories: (1) questions about cross-sectional specificity, (2) questions about longitudinal specificity, and (3) more complex questions about developmental specificity, which involve questions about bidirectional associations and possible mechanisms for the aggravation of difficulties.

First, as concerns cross-sectional specificity, previous research has demonstrated evidence that direct and indirect aggression are differently associated with gender (boys showing more direct aggression, and girls tending to show slightly more indirect aggression) and with various types of psychological problems (direct aggression being primarily associated with externalizing problems, and indirect aggression with internalizing problems). We know little, however, about whether the same applies to direct and indirect *victimization*. A first purpose of the present study was therefore to test the hypothesis that direct and indirect victimization are also differently associated with gender (direct victimization being more common in boys, and indirect victimization in girls) and with various types of psychological problems.

Second, as concerns longitudinal specificity, there is very little research done. Although both aggression and victimization *in general* have been shown to be risk factors for the development of psychological difficulties, we do not know if direct and indirect forms of aggression and victimization differ in the kind of psychological difficulties that they predict over time. A second purpose of the present study was, therefore, to test whether direct and indirect aggression and victimization are risk factors for different types of psychological difficulties, and whether these associations differ by gender.

Third, there is very little research done on more complex questions about prospective bidirectional associations. Although it is relatively well-documented that victimization *in general* shows bidirectional prospective associations with both externalizing and internalizing problems, we do not know whether direct and indirect victimization differ in this regard. Further, very little is known about prospective bidirectional associations between any form of aggression and psychological difficulties. The third purpose of the present study was therefore to test if there are such prospective bidirectional associations. If the existence of such prospective bidirectional associations can be demonstrated, this represents evidence of potentially important mechanisms for the aggravation of existing problems, in the form of dynamic systems with feedback processes that lead to the emergence and stabilization of pathological patterns. Further knowledge along these lines may be important for both the prevention and treatment of psychological problems.

A common theme to these questions is *specificity*. As to the nature of this specificity, scattered findings in several areas (referred to above) suggest that direct aggression and direct victimization tend to be associated with externalizing problems, whereas indirect aggression and indirect victimization tend to go together with internalizing problems – and that boys show more of the former (i.e., direct forms of aggression and victimization, as well as externalizing problems), whereas girls tend to show more of the latter (i.e., indirect forms of aggression and victimization, as well as internalizing problems). It was therefore hypothesized that direct forms of aggression and victimization are associated primarily with externalizing problems and male gender, whereas indirect forms of aggression and victimization are associated primarily with internalizing problems and female gender, both cross-sectionally and longitudinally.

## Methods

### Participants

The participants were a community sample of all students in two grades of regular school in a Swedish municipality who took part in a two-wave longitudinal study with a one-year interval. At Time 1 (T1), this municipality had around 40,000 inhabitants and five schools with students in Grade 7 (n =532) and Grade 8 (n =520), aged 13–15 years. In order to get data from as many students as possible, the ordinary questionnaire sessions at each school were followed by extra sessions for those who were absent at the first session. Altogether, 992 of the 1,052 students (=94%) participated in the data collection at T1, and of these, there were full data on the PANIBI for 979 adolescents (493 girls, 485 boys, and one participant who did not report gender). At T2, there were PANIBI data for 973 adolescents (498 girls, 472 boys, and 3 participants who did not report gender). There was longitudinal data available for 893 individuals (456 girls and 437 boys), representing 85% of all students who were available for inclusion at T1.

The municipality was fairly representative of Sweden in terms of the proportions of children living with both of their parents, and having a foreign background, although slightly more rural than Sweden as a whole and with a slightly lower mean income level and a lower educational level (for details, see Lundh et al. 2008).

### Instruments

*The Positive and Negative Interpersonal Behaviors Inventory* (PANIBI) was first tested in a pilot study with a convenience sample of 296 adolescents, aged 14–15 years, from schools in southern Sweden (Lundh: The Positive and Negative Behaviors Inventory, unpublished). On the basis of this pilot study, the instrument was revised to improve its psychometric properties. One reason for developing the PANIBI was to have an instrument for the measurement of aggression and victimization where the item contents are kept constant across aggression and victimization, so that the same behaviors can be studied from the perspectives of both aggressor and victim.

A further rationale for the development of the instrument was to facilitate the truthful report of aggressive behavior. Björkqvist (1994) questioned especially whether indirect aggression can be validly measured by self-report, arguing that “[s]ince indirect means of aggression are used exactly in order to cover one’s harmful intentions, self-reports of indirect aggression are not likely to be honest” (p. 183). Our assumption was that the truthful self-report of aggressive behavior may be facilitated (a) if participants are first asked about the aggressive behavior that they experience from others, before they are asked about their own aggressive behavior towards others (thus reducing the risk that the respondents become “defensive” when confronted with questions about their own aggressive behavior), and (b) if the questions about aggressive behavior are balanced by questions about prosocial behaviors (thus reducing the focus on aggressive behavior). For this reason, PANIBI is constructed symmetrically along two dimensions: valence (positive vs. negative) and direction of behavior (self-to-others vs. others-to-self).

The PANIBI contains forty statements, asking the general question “How often does it happen that…?”, with a five-point response format ranging from 1 (“never”) to 5 (“very often”). Part A asks 20 questions on the theme “How are you treated by others?”, half of which refer to being the object of others’ aggressive behaviors, and half to being the object of prosocial behaviors from others. Part B contains the mirror image of these questions, on the theme “How do you treat others?” Six subscales are computed: Direct Aggression (DA; 5 items), Indirect Aggression (IA; 5 items), Victim of Direct Aggression (VDA; 5 items), Victim of Indirect Aggression (VIA; 5 items), Treated Well by Others (TWO; 10 items), and Treating Others Well (TOW; 10 items). (See Appendix 1 for the item contents). In the present study only the aggression and victimization scales from PANIBI are used. All PANIBI scales show good internal consistency, with Cronbach’s alpha ranging from α = .70 to α = .86. Test-retest reliability, as calculated on the basis of the pilot study, with a test-retest interval of 44 days was also good: DA *r* = .86; IA-4^a^*r* = .84; VDA *r* = .71; VIA *r* = .80.

*The Strengths and Difficulties Questionnaire – self-report version* (SDQ-s; Goodman 2001). The SDQ-s is a brief psychiatric screening instrument for children and adolescents consisting of 25 items, which make up five 5-item subscales assessing Conduct Problems, Hyperactivity–Inattention, Emotional Symptoms, Peer Problems, and Prosocial Behavior. The participants are instructed to respond to each item on the basis of how things have been for them during the last six months, with three response alternatives: “Not true”, “Somewhat true”, and “Certainly true”. The items are scored 0 for “not true”, 1 for “somewhat true” and 2 for “certainly true”. The SDQ-s has shown good discriminatory ability in detecting disorders diagnosed through standardized semi-structured interviews (Goodman and Scott 1999; Klasen et al. 2000). The factor structure, normative scoring, and psychometric properties of the SDQ-s have been investigated in samples from various countries in Europe, America, and Asia (e.g., Du et al. 2008). The Swedish version of the SDQ-s was validated by Lundh et al. (2008). In the present study we used SDQ Emotional Symptoms as a measure of internalizing problems, and SDQ Conduct Problems as a measure of externalizing problems.

### Procedure

Contact was established with school-managements via head-masters who gave consent to their schools’ participation in the study. Contacts were also established with representatives from school health care in the municipality to facilitate procedures if serious psychological problems or other circumstances related to participants would warrant an intervention. Informed consent was obtained by sending written information to the parents of all students and by handing out information directly to all students in school. This information described the study and stated that participation was voluntary, that all answers were treated confidentially, and that no school personnel would have access to the answers. The students and their parents were informed that they could refrain from participation by telling their respective teachers or by contacting the researchers directly. This passive consent procedure was regarded as the most appropriate means of collecting informed consent in the present context (Celio et al. 2003). The research procedure, including the passive consent procedure, was approved by the Regional Ethics Committee at Lund University (Dnr 49/2006).

The instruments used in the present study were part of an 11-page questionnaire, which also contained other questions not relevant for the present study. All participants filled out the questionnaires in school, as part of a separate lecture hour. The questionnaires were administered by research assistants from Lund University, who were either licensed psychologists or advanced level students of psychology. A teacher was present, but did not participate in the data collection. The students were instructed to answer all questions as best they could, but not to think too much about any answer. They were instructed not to write their names on the questionnaire. Each questionnaire had a code number to make it possible to match the students’ answers on two test occasions, while preserving the confidentiality of their data. If the students had difficulty understanding anything in the questionnaire, they were instructed to raise their hands, and the research assistant then provided individual help. After the completion of the questionnaire they were instructed to seal it in an envelope and then to raise their hands; the research assistant then collected their envelope and gave the student an extra task to work on for the rest of the lecture hour.

### Data analysis

Gender comparisons were done by independent *t-*test and chi-square. Cross-sectional associations were analyzed by Pearson correlations, with Bonferroni correction. To examine the unique relation of each form of aggression and victimization (e.g., direct aggression after controlling for indirect aggression, and indirect aggression after controlling for direct aggression) to the broad categories of psychological difficulties measured by the SDQ, we followed Card et al. (2008) in computing semipartial correlations (*sr*). Differences in the strengths of correlations between boys and girls were tested by converting the two correlations to *z*-scores, dividing the difference between the z-scores with the standard error of difference between the two correlations, and then testing the significance of the *z* value of the difference score.

The one-year stability of the PANIBI scales was computed by Pearson correlations. To analyze whether high values on some variables (e.g., direct and indirect victimization) at T1 were risk factors for the development of other kinds of problems at T2 (e.g., emotional symptoms), hierarchical multiple regressions were used, where the values of the latter variable (i.e., emotional symptoms) at T1 were entered at Step 1, the predictor variables (i.e., direct and indirect victimization) were entered at Step 2, and the interaction of the predictor variables with gender were entered at Step 3. Because the scores on the aggression and victimization scales at both T1 and T2 were skewed and leptokurtic, logarithmic transformations were conducted, resulting in acceptable normal distributions, before entering these variables into the equations.

## Results

### Gender comparisons

The results of the gender comparisons were clearly in line with what was expected on the basis of previous research. As seen in Table 1, gender differences were found on all PANIBI scales, except indirect aggression (IA). Boys scored significantly higher than girls on both direct aggression (DA) and being the victim of direct aggression (VDA). Although the girls scored significantly higher than the boys on being the victim of indirect aggression (VIA), however, they showed only a weak non-significant tendency to score higher than boys on indirect aggression (*p* > .10).

**Table 1:** **Means (and SD) for girls and boys on the PANIBI aggression and victimization scales at T1**

	**Girls**	**Boys**	***df***	***t***	***d***
Direct aggression	1.24 (0.36)	1.42 (0.46)	976	−6.9*	−0.44
Indirect aggression	1.41 (0.45)	1.36 (0.44)	975	1.6	0.11
Victim of direct aggression	1.48 (0.59)	1.58 (0.56)	974	−2.7*	−0.17
Victim of indirect aggression	1.83 (0.80)	1.66 (0.73)	975	3.6*	0.22

*p < .05/4 = .0125.

Table 2 shows the percentages of girls and boys who reported having engaged in the various forms of aggressive behaviors (by responding “seldom”, “sometimes”, “often”, or “very often). The most commonly reported behavior, both in girls and boys, was “saying mean things about someone”. Boys reported more of all forms of directly aggressive behaviors, although the difference on “writing mean things about someone” was not significant when Bonferroni corrections were made. Notably, girls reported more only of one single form of indirectly aggressive behavior: “speaking ill of somebody behind their back”.

**Table 2:** **Percentages of girls and boys who reported having engaged in various forms of aggressive behaviors**

	**Girls**	**Boys**	***χ*** ^**2**^ **(1)**
DA. Hitting or kicking someone	19.3	46.4	81.7*
DA. Yelling negative words at someone	31.3	48.5	30.0*
DA. Giving someone ugly names	24.7	33.4	9.0*
DA. Taking things from someone	9.4	17.4	13.6*
DA. Writing mean things to someone	12.3	18.6	7.2
IA. Saying mean things about someone	52.1	52.8	0.0
IA. Trying to make others dislike someone	19.9	20.7	0.8
IA. Spreading untrue or mean rumours about someone	10.0	13.0	2.2
IA. Ignoring someone, or treating him/her like thin air	34.0	29.4	2.4
IA. Speaking ill of somebody behind their back	45.0	29.4	25.5*

*p < .05/10 = .005.

Table 3 shows the percentages of girls and boys who reported having been a victim of the various forms of aggressive behaviors (by responding “seldom”, “sometimes”, “often”, or “very often). It may be noted that the gender differences are “reversed” here, in the sense that the girls reported more of all forms of indirect victimization, except “somebody says mean things about you”, whereas the boys reported more only of one single form of direct victimization: “somebody hits or kicks you”.

**Table 3:** **Percentages of girls and boys who reported having been the victim of various forms of aggressive behaviors**

	**Girls**	**Boys**	***χ*** ^**2**^ **(1)**
VDA. Somebody hits you or kicks you	22.2	43.8	52.0*
VDA. Somebody yells negative words at you	53.3	62.0	7.6
VDA. Somebody gives you ugly names	45.4	43.7	6.3
VDA. Somebody takes things from you	34.5	38.1	1.4
VDA. Somebody writes mean things to you	22.3	21.9	0.0
VIA. Somebody says mean things about you	61.2	62.3	0.1
VIA. Somebody tries to make others dislike you	53.8	41.7	14.2*
VIA. Somebody spreads untrue or mean rumours about you	48.9	38.1	11.5*
VIA. Somebody ignores you, or treats you like thin air	38.1	27.4	12.6*
VIA. Somebody speaks ill of you behind your back	65.1	53.1	14.5*

*p < .05/10 = .005.

### Cross-sectional associations

Table 4 shows the correlations among the PANIBI scales at T1. Strong positive correlations were found between the two aggression scales (DA and IA), and between the two victimization scales (VDA and VIA), respectively. In addition, both victimization scales showed moderate positive correlations with both aggression scales. This was true for both girls and boys. Although the correlation between DA and IA was slightly higher among boys than girls, this difference was not significant. The correlational patterns were very similar at T2, except that the correlation between DA and IA was significantly higher among boys than girls (*r* = .80 vs. *r* = .66, *z* =4.76, *p* < .001).

**Table 4:** **Zero-order correlations between the PANIBI aggression and victimization scales at T1; girls (** ***n*** **=489-494) above the diagonal, and boys below the diagonal (** ***n*** **=477-484)**

	**DA**	**IA**	**VDA**	**VIA**
Direct Aggression (DA)	--	.60*	.46*	.31*
Indirect Aggression (IA)	.67*	--	.37*	.42*
Victim of Direct Aggression (VDA)	.45*	.40*	--	.74*
Victim of Indirect Aggression (VIA)	.43*	.46*	.74*	--

*p < .05/12 = .004.

### Correlations between aggression and SDQ psychological difficulties

As seen in Table 5, and consistent with expectations based on previous research, semipartial correlations controlling for IA showed that DA was uniquely associated with high conduct problems at both T1 and T2. Semipartial correlations controlling for DA showed that IA was uniquely associated with emotional symptoms at both T1 and T2, which is also consistent with expectations based on previous research.

**Table 5:** **Correlations between aggression and SDQ psychological difficulties at T1 and T2 (semipartial correlations within parentheses)**

	**Direct aggression**		**Indirect aggression**	
	**T1**	**T2**	**T1**	**T2**
SDQ Conduct Problems	.50 (**.38**)	.59 (**.43**)	.33 (.04)	.44 (.06)
SDQ Emotional Symptoms	.12 (.01)	.04 (−**.12**)	.18 (**.14**)	.19 (**.22**)

All significant semipartial correlations are marked in bold (*p* < .05/8 = .006).

### Correlations between victimization and SDQ psychological difficulties

As seen in Table 6, semipartial correlations (*sr*) showed that there was a clear and consistent differentiation between VDA and VIA with regard to associations with emotional symptoms. In accordance with hypotheses, VIA but not VDA showed a unique association with emotional symptoms at both T1 and T2. With regard to conduct problems, however, the results were less clear-cut. Although VDA showed a unique association with conduct problems at both T1 and T2, also VIA did so at T1.

**Table 6:** **Correlations between victimization and SDQ psychological difficulties at T1 and T2** (**semipartial correlations within parentheses)**

	**Victim of direct aggression**		**Victim of indirect aggression**	
	**T1**	**T2**	**T1**	**T2**
SDQ Conduct Problems	.35 **(.16)**	.36 **(.19)**	.33 **(.11)**	.32 (.08)
SDQ Emotional Symptoms	.35 (.08)	.33 (.02)	.41 **(.23)**	.44 **(.29)**

All significant semipartial correlations are marked in bold (*p* < .05/8 = .006).

### Longitudinal associations

As seen in Table 7, all PANIBI scales showed a considerable one-year stability from T1 to T2 (stability coefficients ranging from *r* = .51 to .70).

**Table 7:** **Correlations of the PANIBI scales over a one-year period (one-year stability coefficients in bold)**

	**T2 DA**	**T2 IA**	**T2 VDA**	**T2 VIA**
T1 DA	.51*	.39*	.26*	.22*
T1 IA	.36*	.52*	.20*	.27*
T1 VDA	.26*	.21*	.53*	.47*
T1 VIA	.20*	.25*	.46*	.59*

*p < .05/16 = .003.

### Is victimization at T1 a risk factor for the development of aggression at T2?

As seen in Table 7, victimization at T1 showed weak prospective correlations with aggression at T2 (*r*s ranging from .20 to .26). To test Ostrov’s specificity hypothesis (i.e., that direct victimization would predict direct aggression, and indirect victimization would predict indirect aggression), two hierarchical regression analyses were computed. Although both DA-T1 (*β* = .42, *p* < .001) and IA-T1 (*β* = .12, *p* < .01) significantly predicted DA-T2, neither VDA-1 (*β* = .00, *ns*) nor VIA (*β* = .05, *ns*) did so. Similarly, although both DA-T1 (*β* = .14, *p* < .001) and IA-T1 (*β* = .45, *p* < .001) significantly predicted IA-T2, neither VDA-1 (*β* = −.04, *ns*) nor VIA (*β* = .04, *ns*) did so. Hence, there was no support for Ostrov’s specificity hypothesis.

### Is aggression at T1 a risk factor for the development of psychological difficulties at T2?

As seen in Table 8, direct aggression at T1 was found to be a risk factor for the development of conduct problems at T2. However, when the interactions between aggression and gender were introduced at step 3, significant interactions were found for both direct and indirect aggression. When the model was run separately for each gender, *direct* aggression was a significant predictor for increased conduct problems in boys (*β* =0.25, *p* < .001) but not in girls (*β* =0.02, *ns*); on the other hand, *indirect* aggression was a significant predictor for increased conduct problems in girls (*β* =0.12, *p* < .02) but not in boys (*β* = −0.04, *ns*). Also as seen in Table 8, neither of the aggression scales predicted the development of emotional symptoms.

**Table 8:** **Prospective hierarchical regressions, predicting SDQ psychological difficulties at T2 from direct and indirect aggression at T1** ^**a**^

**Variables**		***R*** ^***2***^ ***Δ***	***B***	***SE B***	***ß***	***F-step***
*Predicting SDQ Emotional Symptoms at T2 from Direct and Indirect Aggression at T1*						
Step 1		.39				281.5***
Gender			−0.95	0.12	−.22***	
T1 Emotional Symptoms			0.53	0.03	.52***	
Step 2		.00				1.2
Gender			−0.88	0.13	−.21***	
T1 Emotional Symptoms			0.53	0.03	.52***	
T1 Direct Aggression			−0.78	0.63	−.04	
T1 Indirect Aggression			0.89	0.60	.05	
*Predicting SDQ Conduct Problems at T2 from Direct and Indirect Aggression at T1*						
Step 1		.25				151.3***
Gender			0.20	0.10	.06	
T1 Conduct Problems			0.52	0.03	.50***	
Step 2		.02				13.8***
Gender			0.13	0.10	.04	
T1 Conduct Problems			0.43	0.04	.41***	
T1 Direct Aggression			2.15	0.59	.15***	
T1 Indirect Aggression			0.56	0.52	.04	
Step 3		.01				4.5*
Gender			0.05	0.14	.02	
T1 Conduct Problems			0.43	0.03	.41***	
T1 Direct Aggression			−2.88	1.79	−.20	
T1 Indirect Aggression			3.69	1.58	.26*	
T1 Direct Aggression × Gender			3.29	1.10	.40**	
T1 Indirect Aggression × Gender			−2.16	1.04	−.25*	

*p < .05, **p < .01, ***p < .001.

^a^Results from Step 3 are only reported where significant effects were found.

### Is victimization at T1 a risk factor for the development of psychological difficulties at T2?

As seen in Table 9, indirect victimization at T1 predicted the development of both emotional symptoms and conduct problems at T2, whereas direct victimization showed no significant effects.

**Table 9:** **Prospective hierarchical regressions, predicting SDQ psychological difficulties at T2 from direct and indirect victimization at T1** ^**a**^

**Variables**		***R*** ^***2***^ ***Δ***	***B***	***SE B***	***ß***	***F-step***
*Predicting SDQ Emotional Symptoms at T2 from Direct and Indirect Victimization at T1*						
Step 1		.39				281.5***
Gender			−0.94	0.12	−.22***	
T1 Emotional Symptoms			0.53	0.03	.52***	
Step 2		.02				11.2***
Gender			−0.90	0.12	−.21***	
T1 Emotional Symptoms			0.48	0.03	.47***	
T1 Victim of Direct Aggression			−0.38	0.58	−.03	
T1 Victim of Indirect Aggression			1.90	0.48	.15***	
*Predicting SDQ Conduct Problems at T2 from Direct and Indirect Victimization at T1*						
Step 1		.25				150.6***
Gender			0.20	0.10	.06	
T1 Conduct Problems			0.52	0.03	.50***	
Step 2		.01				6.2**
Gender			0.15	0.16	.04	
T1 Conduct Problems			0.48	0.03	.46***	
T1 Victim of Direct Aggression			0.28	0.51	.02	
T1 Victim of Indirect Aggression			0.94	0.43	.09*	

*p < .05, **p < .01, ***p < .001.

^a^No results from Step 3 are reported because no significant effects were found.

### Are psychological difficulties at T1 a risk factor for the development of aggression at T2?

As seen in Table 10, conduct problems predicted increases in both direct and indirect aggression, whereas emotional symptoms showed no significant effect.

**Table 10:** **Prospective hierarchical regressions, predicting direct and indirect aggression and victimization at T2 from SDQ psychological difficulties at T1** ^**a**^

**Variables**		***R*** ^***2***^ ***Δ***	***B***	***SE B***	***ß***	***F-step***
*Predicting Direct Aggression at T2 from SDQ subscales at T1*						
Step 1		.30				188.4***
Gender			0.02	0.01	.09**	
Direct Aggression			0.60	0.03	.52***	
Step 2		.02				14.0***
Gender			0.02	0.01	.08*	
T1 Direct Aggression			0.51	0.04	.44***	
T1 Emotional Symptoms			−0.00	0.00	−.04	
T1 Conduct Problems			0.01	0.01	.17***	
*Predicting Indirect Aggression at T2 from SDQ subscales at T1*						
Step 1		.31				195.3***
Gender			−0.01	0.01	−.05	
Indirect Aggression			0.65	0.03	.55***	
Step 2		.01				6.2**
Gender			−0.02	0.01	−.06*	
T1 Indirect Aggression			0.58	0.03	.51***	
T1 Emotional Symptoms			−0.00	0.00	−.01	
T1 Conduct Problems			0.01	0.00	.11***	
*Predicting Direct Victimization at T2 from SDQ subscales at T1*						
Step 1		.29				179.7***
Gender			0.01	0.01	.02	
Victim of Direct Aggression			0.56	0.03	.54***	
Step 2		.02				6.7***
Gender			0.01	0.01	.04	
T1 Victim of Direct Aggression			0.50	0.03	.48***	
T1 Emotional Symptoms			0.01	0.00	.07*	
T1 Conduct Problems			0.01	0.00	.08**	
*Predicting Indirect Victimization at T2 from SDQ subscales at T1*						
Step 1		.36				249.6***
Gender			−0.03	0.01	−.08**	
Victim of Indirect Aggression			0.61	0.03	.58***	
Step 2		.02				8.9***
Gender			−0.02	0.01	−.07*	
T1 Victim of Indirect Aggression			0.54	0.03	.52***	
T1 Emotional Symptoms			0.01	0.00	.08**	
T1 Conduct Problems			0.01	0.00	.08**	

*p < .05, **p < .01, ***p < .001.

^a^No results from Step 3 are reported because no significant effects were found.

### Are psychological difficulties at T1 a risk factor for the development of victimization at T2?

As seen in Table 10, both conduct problems and emotional symptoms predicted increases in both forms of victimization.

## Discussion

Beyond replicating some previous findings, the present results add several new findings. These results are summarized and discussed below in terms of the three categories of questions that were posed in the introduction: (1) questions about cross-sectional specificity, (2) questions about longitudinal specificity, and (3) more complex questions about bidirectional associations and possible mechanisms for the aggravation of difficulties.

### Cross-sectional specificity

First, with regard to gender, although the boys scored significantly higher on direct aggression, there was only a weak non-significant tendency for the girls to score higher than the boys on indirect aggression. This is clearly consistent with the results from Card et al.’s (2008) meta-analysis. On the basis of their results, Card et al. commented that “the general pattern is of similarities rather than differences among boys’ and girls’ use of indirect aggression” (p. 1204), and raised the question “why the misperception that girls are more indirectly aggressive than boys is so pervasive” (p. 1204). The present study confirms these results with regard to indirect *aggression.* At the same time, however, it suggests that there are larger and less trivial gender differences on indirect *victimization*. The boys reported being more victim of direct aggression than the girls, whereas the girls reported being more victim of indirect aggression than the boys. Further, the gender difference on indirect victimization was larger than that on direct victimization, and the girls scored consistently higher on four of the five items on the indirect victimization scale. This clearly suggests that there are important gender differences at least on being victim to indirect aggression that needs to be focused on in future research, as a more detailed knowledge of these processes may be important to understand the development of girls’ psychological difficulties.

The present results also confirm Card et al.’s (2008) finding that direct and indirect aggression show higher correlations in boys than in girls, although this difference was statistically significant only at T2 in our data. As formulated by Card et al. (2008), this suggests that “boys who enact one form of aggression may be inclined to also enact other forms of aggression, more so than for girls” (p. 1207). This contributes to an overall picture that there are real gender differences in this area, which are still insufficiently understood.

With regard to the associations with psychological difficulties, the cross-sectional results from the present study unambiguously support the specificity hypothesis with regard to emotional symptoms, both in relation to aggression and victimization. That is, emotional symptoms showed a consistent and unique association not only with indirect aggression but also with indirect victimization, whereas it did not show any such association with direct aggression or with direct victimization. This suggests that anxious and fearful adolescents are not only more likely to engage in subtle indirect forms of aggression, but also more likely to be the victim of such subtle forms of aggression.

The results, however, are not equally clear-cut with regard to conduct problems. As expected, conduct problems showed a consistent and unique association with direct aggression, which was not found with indirect aggression. But with regard to victimization, conduct problems did not only show a consistent and unique association with being the victim of direct victimization but also tended to show such an association with being the victim of indirect aggression. This means that the specificity hypothesis did not receive unambiguous support with regard to conduct problems. These results imply that, although more of conduct problems is associated specifically with more of direct (and not indirect) aggression towards others, it may be associated with being more a victim of both direct and indirect aggression from others.

In this context, it should be noted that there is a definite conceptual overlap between the PANIBI direct aggression scale and the SDQ measure of conduct problems. The SDQ subscale Conduct Problems contains 5 items, of which at least three refer to aggressive behaviors: “I get very angry and often lose my temper”; “I fight a lot. I can make other people do as I want”; and “I take things that are not mine from home, school or elsewhere”. This means that the associations that were found between direct aggression and conduct problems may be at least partly due to conceptual overlap. The significance of these cross-sectional associations is therefore uncertain.

To summarize: although the differentiation between direct and indirect victimization has sometimes been questioned (e.g., Card and Hodges 2008), the results of the present research indicate that it is definitely empirically meaningful to distinguish between direct and indirect victimization. With regard to gender the distinction between direct and indirect victimization actually seems to be even more relevant than the distinction between direct and indirect aggression. Also with regard to emotional problems the distinction between direct and indirect victimization seems to be equally meaningful as the corresponding distinction between direct and indirect aggression – more of emotional problems is associated specifically with more of indirect aggression towards others and more of indirect aggression from others. On the other hand, the results are less clear-cut with regard to conduct problems.

### Longitudinal specificity

Although direct and indirect aggression, as well as direct and indirect victimization, showed very clear evidence of specificity in the correlational analyses, the picture of the prospective associations given by the longitudinal analyses is more complex. As to aggression, the only clear longitudinal evidence of specificity in the prospective associations of direct and indirect aggression was in the prediction of conduct problems one year later. The interaction between gender and type of aggression that was found in these analyses suggest that whereas only *direct* aggression is a risk factor for the development of conduct problems in boys, only *indirect* aggression is a risk factor for the development of conduct problems in girls. Again, this suggests that it may be premature to conclude, as done by Card et al. (2008), that the gender differences in this area are “trivial”.

A similar difference between boys and girls with regard to the *concurrent* associations of relational aggression with externalizing problems was discussed by Prinstein et al. (2001), who suggested that girls’ conduct problems are more associated with telling rumours and excluding peers from activities than boys’ conduct problems. Different *prospective* associations of the kind that were found in the present study, however, seem to require a more “dynamic” explanation, as they refer to differences in developmental processes. A better understanding of these processes probably requires more detailed research into how boys’ and girls’ profiles of conduct problems may differ, and how different patterns of aggressive behavior are developmentally associated with different patterns of conduct problems.

Concerning the other aspects of the prospective associations between aggression and psychological difficulties in the present study, *neither* direct nor indirect aggression turned out to be risk factors for the development of emotional symptoms. And in terms of reciprocal associations, although conduct problems was found to predict the development of *both* direct and indirect aggression, emotional symptoms showed no such associations. That is, against the hypotheses, there was no evidence of any direct/indirect specificity in these results.

As to the conceptual overlap between the PANIBI direct aggression scale and the SDQ measure of conduct problems that was discussed in the previous section, it is important to note that this does *not* represent any threat to the validity of the conclusions from the longitudinal analyses of the associations between aggression and conduct problems. In the hierarchical regression for predicting T2 Conduct Problems, for example, T1 Conduct Problems was entered together with T1 Direct Aggression as independent variables at step 2, and the PANIBI measures of Direct Aggression was found to contribute to the prediction of T2 Conduct Problems even when T1 Conduct Problems was controlled for (see Table 8); this result can only be due to the *non-overlapping* parts of these measures. In the same way, in the hierarchical regression for predicting T2 Direct Aggression, T1 Direct Aggression was entered together with T1 Conduct Problems as independent variables at step 2, and T1 Conduct Problems was found to contribute to the prediction of T2 Direct Aggression even when T1 Direct Aggression was controlled for (see Table 10); this result also can only be due to the *non-overlapping* parts of these measures.

With regard to victimization, the results indicate that *indirect* victimization but not direct victimization is a risk factor for the development of emotional symptoms. Although this is clearly consistent with the hypotheses, however, exactly the same pattern (against the hypotheses) was found with regard to the prediction of conduct problems – that is, *indirect* but not direct victimization was found to be a risk factor also for the development of conduct problems. Finally, in terms of reciprocal associations, and against the specificity hypothesis, both types of psychological difficulties (conduct problems and emotional symptoms) were found to be prospectively associated with the development of both direct and indirect victimization.

The finding that only indirect but not direct victimization was a risk factor for the development of psychological difficulties may seem surprising. It has to be remembered here that the forms of direct victimization that were asked for in the present study referred to being the victim of hitting, kicking, stealing, and various forms of verbal abuse, whereas there were no questions about more severe forms of physical and sexual abuse. Still, it is interesting that the indirect forms of victimization (including experiences of others speaking ill of you behind your back, spreading untrue or mean rumors about you, ignoring you or treating you “like thin air”, and trying to make others dislike you), which could hardly be said to involve more serious forms of abuse than the direct forms of victimization that were asked about, turned out to predict the development of both emotional symptoms and conduct problems. One possibility is that there is something about the *indirect* nature of this form of aggression (e.g., the treacherous aspects of it) that has particularly damaging consequences for those who become victim of it – for example, it is possible that it may be more damaging to one’s ability to trust others (i.e., you can’t trust what they say to you, because they may do something quite different behind your back).

It should also be noted that the specific prospective associations between similar forms of victimization and aggression that were reported by Ostrov (2008, 2010) in small children were not found among the adolescents in the present sample. One possible interpretation of these results is that “children’s retaliation in kind” does not apply to adolescents in the same way as it does to young children. In fact, victimization did not show up as a risk factor at all for the development of aggressive behavior.

To summarize: With regard to developmental specificity, perhaps the most interesting finding in these analyses was the interaction between gender and type of aggression in the prediction of conduct problems one year later. Whereas direct but not indirect aggression was such a predictor in boys, indirect but not direct aggression was so in girls. This suggests that conduct problems in boys and girls may follow different developmental patterns. In terms of developmental specificity, it is also interesting that being the victim of indirect but not direct aggression was a risk factor for the development of both emotional problems and conduct problems one year later. This suggests that indirect forms of victimization, although they may be less conspicuous, should be taken seriously and that their potentially damaging effects should not be underestimated.

### Bidirectional associations

Finally, with regard to bidirectional associations, several examples were found. First, not only did we replicate the previously reported finding of a bidirectional relation over time between victimization and internalizing problems (Reijntjes et al. 2010), but this was done in the form of a more specific bidirectional association between *indirect* victimization and emotional problems. Similarly, the previously reported finding of a bidirectional relation over time between victimization and externalizing problems (Reijntjes et al. 2011) was also replicated, but also here in the form of a more specific bidirectional association between conduct problems and indirect victimization. These results suggest that not only emotional problems but also conduct problems may enter into a vicious cycle with indirect victimization, potentially leaving room for several different forms of escalating problems.

With regard to aggression, the present results show evidence of a bidirectional association between *direct* aggression and conduct problems in boys, and between *indirect* aggression and conduct problems in girls. This gender-specific differentiation between direct and indirect aggression in their bidirectional associations with conduct problems has, to our knowledge, not been reported in previous research. If replicated in other samples, and by means of other instruments (including observer measures), this may indicate different developmental pathways to antisocial problems among girls and boys.

### Strengths and limitations

The present study has several strengths: it uses a large representative sample of adolescents, and there are longitudinal data for 85% of this sample of adolescents. The study, however, also suffers from several limitations. For example, the study relies on only two assessment points, and it is possible that other results on prospective associations would have been obtained if the first assessment point had been earlier, or if more assessment points had been added. The inclusion of three or more assessment points would have provided more information about the patterns of change over time, which may be especially important if different psychological problems develop differently over time. Another limitation is that the study relies entirely on self-assessment instruments; a multi-method approach with observer measures might have made it possible to draw stronger conclusions, both with regard to validity and prospective associations.

A caveat is that the present study made use of ten hierarchical regressions. Although most of the discussed findings are significant at *p* < .001 and would remain even with a strict Bonferroni correction, some findings are only significant at *p* < .05, and should therefore be interpreted with caution. For example, this applies to the interaction that was found between gender and type of aggression in the prediction of conduct problems one year later. Reducing the alpha level would reduce the risk of reporting false positives, but at the expense of an increased risk of producing false negatives. In view of the exploratory character of the present study, we thought it is more important not to miss a potentially important finding than to guard against false positives. At the same time, however, the need for caution in interpreting the present findings has to be emphasized. For example, this means that the interaction that was found between gender and type of aggression in the prediction of conduct problems needs replication before any more definite conclusions are drawn.

It is also important to remember that, although longitudinal studies (by showing evidence of temporal order) are far more powerful than cross-sectional ones in the search for possible causal relationships, they still do not permit any strong causal inferences. Although the present results indicate, for example, that indirect victimization predicts increases in emotional problems, and vice versa, it might still be the case that increases in both of these variables may be explained by changes in some third variable (e.g., other kinds of adverse life events, or the absence of positive events).

There may also be other aspects of aggression than those studied here that are important to take into account in future research on the developmental implications of direct and indirect aggression. For example, Ostrov et al. (2013) recently reported that the prospective associations of relational aggression in early childhood went in opposite directions depending on whether the aggression was reactive or proactive; proactive relational aggression was associated with decreases in peer rejection, whereas reactive relational aggression was associated with increases in peer rejection over time. This suggests that future research on indirect aggression in adolescents may benefit by including measures of the reactive and proactive functions of aggressive behavior.

Finally, it should be noted that although most previous research on victimization has had a specific focus on peer victimization, this study makes no distinction between victimization by peers and others. Although this is not necessarily a limitation of the present study, this difference should be kept in mind when comparing these results with those from other studies.

## Conclusions

One main contribution of the present study is that it gives a more detailed picture than previous research of potentially important differences between direct and indirect aggression, and between direct and indirect victimization. By the use of a prospective, longitudinal design, the present findings show that direct and indirect aggression, as well as direct and indirect victimization, may have different roles in the development of psychological problems in young adolescents.

With regard to aggression, the present results suggest that boys and girls differ not only in their tendency to engage in direct versus indirect aggression, but also that direct and indirect aggression seem to have different developmental consequences in boys and girls. Whereas *direct* aggression was uniquely associated with an increase in conduct problems among boys (but not among girls), *indirect* aggression was uniquely associated with an increase in conduct problems among girls (but not among boys).

With regard to victimization, the present results show that direct and indirect forms of victimization are differently associated with gender and type of psychological difficulties, and that indirect victimization may be an important risk factor for the development of both internalizing and externalizing problems.

Further, the demonstration of prospective bidirectional associations involving both aggression and victimization points to possible mechanisms for the development of psychological difficulties that may be described in terms of dynamical systems theory. This has potential relevance both for the prevention and the treatment of psychopathology.

## Endnote

^a^In the pilot study, an earlier version of the IA subscale was used, which contained an item (“How often does it happen that you exclude others from groups?”) that showed a weak item-total correlation (*r* = .19), resulting in a low internal consistency of the IA subscale (α = .58). When this item was excluded the alpha value of this subscale (here referred to as IA-4) increased to α = .64; test-retest reliability was calculated for this 4-item subscale. On the basis of additional pilot work, this problematic item was later revised into “How often does it happen that you try to make others dislike someone?”, which led to a five-item IA scale with good internal consistency (α = .77).

## Appendix 1

Items in the PANIBI (VDA = Victim of Direct Aggression; VIA = Victim of Indirect Aggression; DA = Direct Aggression; IA = Indirect Aggression; TWO = Treated Well by Others; TOW = Treating Others Well)

***A. How often does it happen***

1. that somebody hits you or kicks you? (VDA)

2. that somebody gives you a hug? (TWO)

3. that somebody yells negative words at you? (VDA)

4. that somebody gives you praise? (TWO)

5. that somebody says mean things about you? (VIA)

6. that somebody invites you to a party? (TWO)

7. that somebody tries to make others dislike you? (VIA)

8. that somebody listens with interest to what you have to say? (TWO)

9. that somebody spreads untrue or mean rumours about you? (VIA)

10. that somebody helps you with difficult tasks? (TWO)

11. that somebody ignores you, or treats you like thin air? (VIA)

12. that somebody smiles at you or gives you appreciative glances? (TWO)

13. that somebody speaks ill of you behind your back? (VIA)

14. that somebody asks for your opinion? (TWO)

15. that somebody gives you ugly names? (VDA)

16. that somebody gives you presents? (TWO)

17. that somebody takes things from you? (VDA)

18. that somebody sends positive messages to you? (TWO)

19. that somebody writes mean things to you? (VDA)

20. that somebody says positive things about you to others? (TWO)

***B. How often does it happen***

1. that you hit or kick somebody? (DA)

2. that you give someone a hug? (TOW)

3. that you yell negative words at someone? (DA)

4. that you give someone praise? (TOW)

5. that you say mean things about someone? (IA)

6. that you invite someone to a party? (TOW)

7. that you try to make others dislike someone? (IA)

8. that you listen with interest to what someone say? (TOW)

9. that you spread untrue or mean rumours about someone? (IA)

10. that you help someone with difficult tasks? (TOW)

11. that you ignore someone, or treat him/her like thin air? (IA)

12. that you give someone a smile or appreciative glances? (TOW)

13. that you speak ill of somebody behind their back? (IA)

14. that you ask someone for their opinion? (TOW)

15. that you give someone ugly names? (IA)

16. that you give presents to someone? (TOW)

17. that you take things from someone? (DA)

18. that you send positive messages to somebody? (TOW)

19. that you write mean things to somebody? (DA)

20. that you say positive things about someone to others? (TOW)

## Abbreviations

DA: Direct aggression
IA: Indirect aggression
PANIBI: Positive and negative interpersonal behaviors inventory
SDQ: Strengths and difficulties questionnaire
VDA: Victim of direct aggression
VIA: Victim of indirect aggression
